# Midwives’ knowledge and practices regarding the screening for and management of chorioamnionitis: A qualitative study

**DOI:** 10.4102/hsag.v26i0.1631

**Published:** 2021-11-09

**Authors:** Allison H. du Plessis, Dalena van Rooyen, Wilma ten Ham-Baloyi

**Affiliations:** 1 Faculty of Health Science, Nelson Mandela University, Port Elizabeth, South Africa

**Keywords:** practices, knowledge, chorioamnionitis, screening and management, midwives

## Abstract

**Background:**

Screening for chorioamnionitis, or the risk thereof, by midwives is largely lacking during antenatal care and no best practice guidelines for chorioamnionitis in South Africa was noted.

**Aim:**

To explore and describe midwives’ knowledge and practices related to the screening and management of women who are at risk of or diagnosed with chorioamnionitis.

**Setting:**

Public healthcare institutions in a health district in the Eastern Cape province of South Africa.

**Methods:**

A qualitative, exploratory, descriptive and contextual research design was used. Ten midwives were purposively included in this study, and semi-structured interviews were conducted with them. The data were analysed using an adapted version of Tesch’s eight steps for data analysis.

**Results:**

The main theme revealed that midwives lack knowledge regarding chorioamnionitis, resulting in incorrect practices including a lack of screening, misdiagnosis and mismanagement of the infectious condition.

**Conclusions:**

Findings of this research showed that midwives lacked knowledge regarding the screening and management of women with chorioamnionitis resulting in incorrect practices in this regard. There is thus a need for midwives to update their knowledge regarding the screening and management of chorioamnionitis and training (e.g. through a short learning programme).

**Contribution:**

Findings of this study could be used by midwives to update their knowledge regarding screening and managing women with chorioamnionitis, which is expected to translate to better practices. Moreover, study findings were synthesised with the results of a literature review study to form the basis for the development of a best practice guideline for screening and managing women with chorioamnionitis.

## Introduction

The neonatal death rate has generally declined globally by more than half from 1990 to 2018 (from 37/1000 to 19/1000 live births), yet neonatal deaths account for 47% of all under-5 child mortality (World Health Organization [WHO] 2019a). Ninety-eight per cent of new-born mortality occurs in low- and middle-income countries (LMICs), of which 78% occur in sub-Saharan Africa and Asia. The Saving Babies Report revealed that 2.2% of the neonatal deaths in South Africa are due to pregnancy-related infections, and 48.0% of unexplained intra-uterine deaths occurred in women with no obstetric complications (South Africa National Department of Health 2017).

Perinatal infection was reported by the WHO to be the third-highest death factor for maternal mortality rates in the world in 2013, and United Nations Children’s Fund (UNICEF) reported that primary causes of new-born deaths include premature births (35%), complications around the time of birth 24%), and infections such as sepsis and meningitis (15%) and pneumonia (6%) (UNICEF 2018). Inclusive of these infections is chorioamnionitis, which is defined as an inflammation (elicited by an immune response) or infection (elicited by bacterial infection) of the foetal sac, and is a cause of neonatal infections (El Malek, Embleton & Loughney 2015; Gantert et al. 2010).

A new name for chorioamnionitis was suggested by the National Institute of Child Health and Human Development expert panel: ‘Intrauterine inflammation or infection or both’, abbreviated as ‘Triple I’, which has been proposed to include all the parameters of chorioamnionitis that involve the amniotic fluid, foetus, umbilical cord or placenta as well as the foetal membranes (Peng et al. 2018). The inflammation or infection may extend to the placenta and the uterine muscle in more severe cases.

Chorioamnionitis is caused by ascending infections such as organisms that cause vaginitis, streptococcal infections (in the form of a urinary tract infection [UTI]) and other underlying infections, which ascend the vaginal tract to affect the foetal membranes. Allen and Yudin (2012) report that UTIs caused by the bacteria *Escherichia coli, Ureaplasma urealyticum* and group B streptococci were highlighted in research studies as culprits in ascending bacterial infections that cause chorioamnionitis. UTIs, bacterial vaginitis and other sexually transmitted infections, which are part of the burden of disease in South Africa, and a concern in pregnancy, were also culprits in ascending bacterial infection causing premature deliveries, maternal infections and intra-amniotic infections (Brocklehurst et al. 2013).

Clinical studies in South Africa emerged from the study of placentas of babies born with low Apgar scores. Moore, Arnold and Wright (2013) examined 72 placentas of premature infants who had surgery for necrotising enterocolitis. The results suggested that antenatal placental infection contributed to the pathology of necrotising enterocolitis of neonates.

Identifying intrapartum intraamniotic infection early and implementing treatment recommendations are essential steps to minimise morbidity and mortality for women and new-borns (Committee on Obstetric Practice 2017). Timely maternal management, together with notification of the neonatal healthcare providers, will facilitate appropriate evaluation and antibiotic treatment when indicated. Intraamniotic infection alone is rarely, if ever, an indication for caesarean delivery. Complications of chorioamnionitis include post-partum haemorrhage and uterine sepsis. Neonates who were exposed to chorioamnionitis showed signs of respiratory distress syndrome and post-partum infections, such as lung infections (Jones et al. 2013).

Maternal complications such as chorioamnionitis, premature birth and intra-uterine deaths are higher among populations within the lower socioeconomic and educational levels (WHO 2019b). The long-term health effects of chorioamnionitis and its complications have a negative impact on vulnerable socioeconomic populations who are at risk, and it escalates the healthcare cost related to treating the long-term health effects (Ventres, Dharamsi & Ferrer 2017). Anecdotal evidence, observed by obstetricians in a health district in the Eastern Cape province, South Africa, of an increase in hysterectomies in young women due to misdiagnosis of chorioamnionitis motivated the inquiry into screening for and management of women with chorioamnionitis.

Statistics for chorioamnionitis were not monitored in South Africa via the District Health Information System. Thus, the rates of occurrence of chorioamnionitis were assumed by healthcare professionals through inductive reasoning, based on commonly occurring complications of chorioamnionitis, such as premature birth (for unspecified reasons) and what, anecdotally, appeared to be high rate of post-partum removal of uteri because of complications related to infection after a normal vertex delivery. However, hospital patient records revealed that women who presented with a clinical picture that suggested chorioamnionitis were not referred. The complex nature of diagnosing chorioamnionitis further caused uncertainty amongst midwives over the management of these women.

Anecdotal evidence from healthcare professionals in a health district in the Eastern Cape province, South Africa, as observed by the first author, suggests that screening for risks of chorioamnionitis was lacking during antenatal care. Missing risks for chorioamnionitis would result in misdiagnosis and failure to provide timeous treatment to prevent maternal and neonatal complications (Committee on Obstetric Practice 2017). When searching for best practice guidelines on the screening and management of chorioamnionitis, none were found. This, it was assumed, added to the lack of comprehensiveness of the information on the management of chorioamnionitis in the *Guidelines for Maternity Care in South Africa* (South Africa National Department of Health 2015), which is the principal guideline used by midwives in primary health care.

The objective of the study was to explore and describe midwives’ knowledge and practices related to screening for and management of women who are at risk of or who are diagnosed with chorioamnionitis.

## Design

A qualitative research design was chosen for this study as little was known about midwives’ knowledge and practices relating to the screening and management of women who are at risk of or diagnosed with chorioamnionitis. The study aimed to produce knowledge and understanding of the knowledge and practices of midwives by conducting semi-structured individual interviews with them regarding screening and managing women who are at risk of chorioamnionitis and those who present with signs and symptoms of chorioamnionitis and who were therefore diagnosed with chorioamnionitis. An explorative, descriptive and contextual design was chosen as suitable for this study. The philosophical approach was based on the epistemic view of presumptions, and how it relates to arguments from a position of lack of appropriate knowledge (Bodlović 2019). The view that presumptions are inferred from basic facts – in this case, basic experience in the field of midwifery – provides a template for the belief that midwives would speak honestly and truthfully (sometimes called the ‘presumption of veracity’) (Bodlović 2019:580) about how they screen and manage chorioamnionitis.

## Methods

The setting, study population and sampling strategy, data collection, data analysis, ethical considerations and steps taken to ensure the quality of research will now be discussed.

### Setting

The research was conducted in a large health district in the Eastern Cape Province, South Africa, in public healthcare institutions.

The health district where the study was performed has a 52.1% poverty rate, a 26.8% unemployment rate, and 3.5% of school-age children are not attending school (South Africa Eastern Cape Department of Health 2015), indicating lower socioeconomic and educational levels. The institutions in this health district mainly serve populations and communities where the prevalence of HIV, sexually transmitted infections, pregnancies, premature rupture of membranes and premature births is high – all of which could be predisposing factors associated with chorioamnionitis.

The midwife obstetric units (MOUs), two public hospitals, Community Health Centres (CHCs) and primary healthcare clinics (PHCs) were targeted for participation in this study. These public healthcare settings vary regarding structure, resources (including the availability of medical supplies, equipment, and human resources) and are providing various levels of care. The maternity care environments in MOUs are usually perceived as stressful by midwives, as they are practising without the presence of medical practitioners. In contrast, midwives at the referral hospitals could directly consult with medical practitioners and discuss the management of patients.

### Study population, sampling and recruitment

The total midwife population in the public health sector in question was 156. Purposive sampling was used according to the following criteria in order to select participants who best could answer the interview questions: midwives must have worked at MOUs, public hospitals, CHCs and PHCs for at least 2 years and were willing to participate. The researcher provided the midwives employed at the selected facilities (the MOUs, two public hospitals, CHCs and PHCs) with information on the study, including the above-mentioned criteria for potential participants to determine their eligibility, and her contact details. Eligible midwives who were interested to participate were requested to contact the researcher. The sample size was determined by data saturation.

### Data collection

Informed consent for interviews was obtained for all participants that contacted the researcher and a time and place was selected convenient for them. Interviews were conducted at the participants’ workplace or their homes for the sake of privacy and a quiet environment. Four (4) participants were interviewed during on-duty times and six (6) participants were interviewed during off-duty times. Data were collected through face-to-face, semi-structured interviews between March and April 2018.

A patient scenario of chorioamnionitis was developed using evidence-based literature. To ensure that the patient scenario was of a reasonable length to avoid long and cumbersome reading for the participants, principles described by Yin (2017) were adapted to refine the theoretically constructed patient scenario. The scenario was reviewed by two academics with expertise in midwifery and tested in a pilot study where face-to-face semi-structured interviews were conducted with two eligible midwives. When the main interview question was tested during the pilot study, the midwives discussed the scenario instead of what happens in real-life practice. The wording of the main question was then changed so that midwives can relate their daily practices regarding the screening for and managing of chorioamnionitis. The data collected from midwives in the pilot study were not used in the main study.

To elicit truthful information from participants and to gauge whether midwives could identify the condition, the word ‘chorioamnionitis’ was omitted from the patient scenario. The main question and semi-structured questions are presented in Table 1.

**TABLE 1:** Interview schedule for midwives.

The main question	Sub-questions
What is your diagnosis of this patient in the scenario and what do you usually do in a case such as this, to manage the patient?	On what principles do you base your diagnosis? What is done in a case such as this to confirm your diagnosis? What does the management in such cases entail? What is done at your facility to prevent and manage such cases? What guides your practice in such a case?

Note: On summarising the primary interview session, the researcher posed the following additional questions that were unrelated to the scenario to the midwives:

What is your opinion regarding The Guideline for Maternity Care in South Africa (2015) on chorioamnionitis?

What suggestions regarding the management of chorioamnionitis do you propose for a best practice guideline’?

The scenario was presented by the first author to participants at the start of each interview to elicit their knowledge and ability to diagnose and manage for a patient that shows signs and symptoms of chorioamnionitis and was collected from the participants after they made a diagnosis and indicated that they understood the scenario. Probing questions were addressed to the participant until the main question was exhausted. The sub-questions were asked if the information was not elicited from the main question. Additional questions, not related to the scenario, were asked to elicit data of midwives on the screening and management of chorioamnionitis, irrespective of their ability to diagnose chorioamnionitis or not (see Table 1).

Data collection was discontinued after data saturation was achieved, which was after 10 interviews. The length of the interviews with midwives ranged between 35 and 110 min. Interviews were digitally recorded on permission from participants and descriptive field notes were taken during the interview sessions to capture the non-verbal data that was noteworthy and included in the transcriptions for complete data. After each interview was conducted, the participant was asked not to discuss the scenario with midwife-colleagues to ensure truthful data was obtained in the following interviews. The first author reflected after each interview session to internalise the interaction with the participants and to perform preliminary data analysis in order to enhance the quality of subsequent interviews and gauge for data saturation.

### Data analysis

The first author repeatedly listened to the digital recordings to capture what the participants said and to become familiar with the content of the interviews. Data were transcribed verbatim and supplemented with the field notes for a better description of the findings. Pseudonyms were assigned to the transcriptions, and the accuracy of the transcribed data was verified by an independent professional transcriber.

An adapted version of Tesch’s eight steps in the coding process, as described by Creswell (2014), was executed in the following five steps: (1) the transcripts were read several times to gain familiarity with the data and to improve the accuracy of understanding of the participants’ perspectives. (2) A list of similar topics was created and grouped together in themes and sub-themes. (3) These were then coded and categorised. (4) A preliminary analysis was performed by grouping each code into categories. (5) Three sessions were held with the co-coder to verify the identified main theme and four sub-themes, as outlined under ‘Findings’.

### Ethical considerations

Ethical clearance was obtained from Nelson Mandela University (reference number: H17-HEA-NUR-006). The researcher adhered to The Researchers Conduct of the university where the study was registered. Permission was obtained from the District Manager, sub-district managers, and the facility managers of the district where the study was conducted. There were no unexpected ethical issues or dilemmas that emerged during the research period. The tenets of the Belmont Report on ethical principles and guidelines for the protection of human subjects in research were followed (United States Government, Belmont Report 1979), which included respect for persons, beneficence and justice. Privacy and confidentiality were ensured by arranging individual interviews in places chosen by the participants. Recorded interviews omitted identifying personal details and replaced them with pseudonyms to ensure anonymity. The verbatim transcriber and co-coder– who were from an independent company outside the research area – committed to confidentiality, based on business principles. All voice recordings, verbatim transcripts and field notes are stored digitally with passwords for a period of 5 years for audit purposes, whereafter data will be destroyed, according to institutional policy.

### Quality of research

The approach to trustworthiness for this research study was adopted from Ravitch and Carl Mittenelner (2016), focusing on criticality, reflexivity and collaboration. To maintain criticality, the researcher acknowledged fallibility, adopted an attitude of learnership and sought to engage, contextualise and make meaning of the complexity of the position of the midwife, with her experience and knowledge, as a practitioner. For the purpose of reflexivity, the researcher reflected upon her identity as an advanced midwifery practitioner with extensive clinical midwifery experience and a vast understanding of the midwifery field. Collaboration was established with experts in the field of midwifery and obstetrics, research supervisors and colleagues to discuss ideas and thoughts regarding the topic, allowing critique and review of the patient scenario. A pilot study was conducted with two midwives. The pilot study with the midwives indicated a need for changing the main question for clarity and therefore the pilot study data was not used in the main study. Main themes and sub-themes were verified by an independent coder.

## Findings

The 10 midwives that participated in the study had between 3 and 22 years of experience in the field of midwifery. The midwives had differing levels of experience and qualifications but had experience related to the antenatal, intrapartum and postpartum care of pregnant women. However, the midwives at PHCs had mainly antenatal and postnatal care experience. Four (*n* = 4) had basic 4-year nursing qualifications and six (*n* = 6) had postgraduate qualifications in advanced midwifery and neonatology. The demographics of the participants are provided in Table 2.

**TABLE 2:** Demographic characteristics of the midwives.

Participant	Years of experience	Qualifications
Annie	14	4-year diploma (general, midwifery, community, psychiatry)
Bettie	22	4-year diploma (general, midwifery, community, psychiatry) + 1-year Diploma in Advanced Midwifery
Cecile	15	4-year diploma (General, midwifery, community, psychiatry) + Honours in Advanced Midwifery
Dali	3	3-year diploma in general + 1-year Diploma in Midwifery
Ester	17	4-year diploma (general, midwifery, community, psychiatry) + 1-year Diploma in Advanced Midwifery
Fred	6	4-year degree (general, midwifery, community, psychiatry) + Masters in Advanced Midwifery
Gerda	4	4-year diploma (general, midwifery, community, psychiatry)
Heather	12	4-year diploma (general, midwifery, community, psychiatry)
Ilse	13	4-year diploma (general, midwifery, community, psychiatry) + Honours in Advanced Midwifery
Jane	8	4-year Bachelor degree (general, midwifery, community, psychiatry) + Masters in Advanced Midwifery

One main theme with four sub-themes that emerged from the data from the interviews with the midwives is set out in Table 3.

**TABLE 3:** Table of themes and sub-themes.

Main theme	Sub-themes
1. Midwives lack knowledge regarding chorioamnionitis, resulting in incorrect practices including a lack of screening, misdiagnosis and mismanagement of the infectious condition.	1.1 There is a lack of screening for chorioamnionitis in antenatal clinics. 1.2 Midwives incorrectly diagnose or misdiagnose chorioamnionitis. 1.3 There is no different management in labour, delivery and the postpartum period for women who were exposed to chorioamnionitis. 1.4 Midwives do not use the guidelines related to the management of chorioamnionitis.

### Main theme: Midwives lack knowledge regarding chorioamnionitis, resulting in incorrect practices including a lack of screening, misdiagnosis and mismanagement of the infectious condition

It was evident from the answers to the questions on the patient scenario that the midwives lack knowledge regarding chorioamnionitis, resulting in incorrect practices including a lack of screening, misdiagnosis and mismanagement of the infectious condition. When they were asked to diagnose the case presented in the patient scenario, only the following four midwives: Ilse, Cecile, Fred and Jane diagnosed chorioamnionitis correctly.

Most midwives diagnosed UTIs based on the leucocytosis presented in the patient scenario or ‘infection’ as a broad diagnosis. The midwives derived this broad diagnosis from the elevated body temperature that was presented in the patient scenario. The following are quotes from midwives when asked to diagnose the patient scenario or to explain what they understood from the scenario.

‘First of all, this patient had a … sorry [*clears her throat*] … I think with a … a raised temperature were infection – leukocytes in the urine.’ (Annie, 14 years experience, midwifery)

Midwives are unfamiliar with chorioamnionitis even though the clinical signs and symptoms were clearly illustrated in the patient scenario. An advanced midwife reiterated the signs and symptoms of chorioamnionitis in the patient scenario yet could not diagnose chorioamnionitis.

‘… with membranes, query rupture of membranes. With a raised temperature, raised pulse, abdominal tenderness with a closed os. CTG also shows slight foetal tachycardia. She’s got leukocytes in the urine. Together with the temperature that would suggest a urinary tract infection.’ (Ester, 17 years experience, midwifery)

When the researcher asked about practices regarding the prevention of chorioamnionitis in antenatal care, midwife Ilse expressed that there is ignorance among midwives in antenatal regarding chorioamnionitis, and that the condition is mis-diagnosed.

‘There’s very little … There’s still ignorance … It [*chorioamnionitis*]’s definitely missed. You just hear UTI.’ (Ilse, 13 years experience, midwifery)

The few midwives who correctly diagnosed chorioamnionitis were advanced midwives, and they diagnosed the condition based on theoretical knowledge about chorioamnionitis.

‘The guidelines will actually give you the scenario that you gave me now. They will tell you that could lead to chorioamnionitis.’ (Ilse, 13 years experience, midwifery)

The four midwives: Ilse, Cecile, Fred and Jane, who could make a correct diagnosis indicated that they had either not managed a patient with chorioamnionitis or they had had a patient with partial symptoms of chorioamnionitis. These midwives acknowledged that it is difficult to diagnose chorioamnionitis if the woman does not present with all the signs and symptoms. They also said that chorioamnionitis is difficult to diagnose when the amniotic sac is intact.

#### Sub-theme 1.1: There is a lack of screening for chorioamnionitis in antenatal clinics

There is no specific practice in the health district reviewed regarding screening for chorioamnionitis, especially in an antenatal clinic. The midwives in both the antenatal clinics and MOUs confirmed that they do not screen for chorioamnionitis and, unlike specific screening practices for diseases such as tuberculosis and HIV, there is no screening tool used to identify factors that could put women at risk for chorioamnionitis. Bettie and Ilse, who had worked for 22 and 13 years, respectively, in the midwifery field, had similar answers when the researcher asked whether screening tools are available for detecting chorioamnionitis.

‘… not that I know of.’ (Bettie, 22 years experience, midwifery)

‘… not that I’m aware of.’ (Ilse, 13 years experience, midwifery)

Participant interviews also revealed that screening for UTIs and vaginal infections is not linked to chorioamnionitis. Routinely on a follow-up visit, the patient is asked about vaginal discharges but is not thoroughly examined to rule out probable causes for ascending infections that could cause chorioamnionitis. There is inconsistency with the type of antimicrobial use for UTIs and vaginosis, despite treatment guidelines being available. Midwives, however, believe that women should not be treated with the same antimicrobials for recurrent UTIs, as explained by the following participant:

‘UTIs shouldn’t be treated across the board with the same antimicrobial for everybody. In my opinion you should do an MC&S [*microscopy, culture and sensitivity*] for every UTI to make sure that you’re using the right antibiotic.’ (Ester, 17 years experience, midwifery)

#### Sub-theme 1.2: Midwives incorrectly diagnose or misdiagnose chorioamnionitis

It is evident from the interviews that midwives incorrectly diagnose chorioamnionitis. One common error is to mistake signs and symptoms of chorioamnionitis with other conditions in pregnancy – for example, UTIs and vaginosis. Midwives confirmed that the women diagnosed and treated for UTI or vaginosis are not followed up after completion of the course of antimicrobials, with the result that when the women return for the next antenatal visit and the UTI or vaginosis is not resolved, more antimicrobials are given, leading to an over-exposure of antimicrobials and resistance for the causative microorganisms, especially when the microscopy, culture and sensitivity (MC&S) are not done or are delayed. Annie confirmed that follow-up after treating a UTI is not done.

‘Even the UTIs is not treated … Sometimes the patient’s got an infection. You give the medication to the patient, but you don’t follow [*up on*] the patient. You don’t give the patient a follow-up date to make sure that that patient took that treatment--that infection [*either*] went away or it got better or it got worse.’ (Annie, 14 years experience, midwifery)

In many cases where a specimen for MC&S is done, the specimen is returned from the laboratory due to contamination. The specimen thus needs to be re-taken, resulting in the delay of provision of the correct treatment. When participants spoke about managing UTIs, one said the following:

‘Most of the time that urine is dirty, when it comes from the lab, they say that specimen is contaminated. So, you must re- take that urine.’ (Dali, 3 years experience, midwifery)

Furthermore, related to incorrectly diagnosing or misdiagnosing chorioamnionitis, midwives confirmed that women with signs and symptoms of chorioamnionitis do not always present with all the clinical symptoms. It is difficult, therefore, to diagnose chorioamnionitis. They also say that it is particularly difficult if the woman presents with intact membranes. It appeared that ruptured amniotic membranes with foul-smelling amniotic fluid are the ultimate symptom that makes it clearer for midwives to diagnose chorioamnionitis. Cecile said the following when asked about diagnosing chorioamnionitis:

‘It’s difficult to say there is chorioamnionitis if the membranes are intact, because there may be other reasons why there is a raised temperature and pulse. So, it’s easy when the membranes are ruptured. It’s really easier. If the membranes is intact, it is to miss it… and then you diagnose something else.’ (Cecile, 15 years experience, midwifery)

Participants admit that they misdiagnose chorioamnionitis. Heather indicated the following regarding the misdiagnosis of chorioamnionitis:

‘It’s missed … because sometimes we think that… maybe out of three symptoms, maybe you will see one or two, and the second one is just slightly, and then you don’t know … So, really, chorioamnionitis is one of those conditions that we missed.’ (Heather, 12 years experience, midwifery)

Ilse answered the following on the same question:

‘… it’s like, when you phone a doctor and you as a midwife you see the symptoms. You know something is wrong. You say, doctor I think its chorioamnionitis. The doctor will tell you; how do you know that? It could be any other infection. Because, the membranes are still intact, you don’t know.’ (Ilse, 13 years experience, midwifery)

#### Sub-theme 1.3: There is no different management in labour, delivery and the postpartum period for women who were exposed to chorioamnionitis

Participants reported that there is no different management during labour, birth and the postpartum period for women who were exposed to chorioamnionitis. Women who are at risk or exposed to chorioamnionitis are receiving the same routine care as women who were not exposed to chorioamnionitis. Women with temperatures between 37.5 and 38 degrees Celsius are given antipyrexial treatment, such as paracetamol tablets and oral antibiotics. The neonate of a pyrexial mother is transferred to the Neonatal Intensive Care Unit if the neonate shows respiratory distress.

As a standard protocol, women with ruptured amniotic membranes and not in labour are kept in the MOUs for 12 h, and those that are in labour are transferred to the referral hospital, with a diagnosis of prolonged rupture of membranes. Midwives working at the referral hospital confirmed that women diagnosed with chorioamnionitis get put on intravenous antibiotics and delivered as soon as possible. Some midwives said that the doctors request observation for 24 h before transfer to the referral hospital if the woman remains undelivered. Midwives do not agree with this practice because they claim that women with ruptured amniotic membranes are put further at risk for chorioamnionitis and get exposed to unnecessary interventions, such as ‘stealing’ a digital vaginal examination (i.e. doing a vaginal examination outside guidelines):

‘ … Before we use to transfer them after 12 hours of rupture of membranes, but now they don’t accept patients [*until*] after 24 hours. Sometimes they develop this thing, chorioamnionitis, while they are still here. The midwives, when they see the patient is having pains, they say: “hey, let me steal vaginal examination”.’ (Cecile, 15 years experience, midwifery)

#### Sub-theme 1.4: Midwives do not use the guidelines related to the management of chorioamnionitis

Midwives admit that they do not use the *Guidelines for Maternity Care in South Africa* (South Africa National Department of Health 2015). They also admit that they do not know what is in these guidelines regarding chorioamnionitis. While midwives spoke about what happens in practice regarding screening and managing women at risk for chorioamnionitis, it was evident that this was done at the discretion of the midwives. When the researcher enquired whether guidelines are followed, Annie said that she is not up to date with the guidelines, stating:

‘To be honest with you, I’m a bit outdated with it [*Guidelines for Maternity Care in South Africa*], honestly.’ (Annie, 14 years experience, midwifery)

Ester admitted that she did not read the guidelines:

‘I’m going to say, I haven’t read it [*Guidelines for Maternity Care in South Africa*].’

Jane is an advanced midwifery practitioner and said the following when the researcher asked her for her thoughts on the guidelines regarding chorioamnionitis:

‘Oh, let me try and remember. I actually, in all honesty, cannot off the top of my hat remember what … the guidelines, what we need to do specifically for chorioamnionitis. I know how we treat it in the ward, but I think a lot of times the guidelines go unused.’ (Jane, 8 years experience, midwifery)

Concerning the same issue, Jane made the following statement:

‘I think that when it comes to things like maternity guidelines, we just get told go read up yourself and a lot of people are lazy to do that….’ (Jane, 8 years experience, midwifery)

Both Fred’s and Jane’s comments indicate a lack of continuous educational development among midwives. With her statement, Jane also implied that there is a lack of in-service training on guidelines.

In different interviews, Ilse referred to her experience regarding telephonic consultation with medical practitioners. She expressed frustration regarding outside-the-guideline orders and that it is difficult to follow the guidelines when medical practitioners give different orders for treating infections. The following quote is characteristic of the confusion and doubts that midwives experience regarding whether to follow the guidelines for chorioamnionitis.

‘… seemingly those guidelines are made for the clinics and the MOUs, because when you… when you discuss these patients you’ll say “Doctor, these guidelines are saying…” and the doctor will say “Those guidelines are not for me, for the hospital. They are for you there in the clinics and MOUs”. So, you, you find yourself, you know, doubting and with the different preferences of the doctors ….’ (Ilse, 13 years experience, midwifery)

Constant telephonic orders outside the guidelines discourage midwives to use the guidelines because they might incorrectly perceive that the guidelines are not important or not applicable. They can also perceive that the guidelines are ineffective because when midwives consult with medical practitioners telephonically they override the guidelines.

## Discussion

Chorioamnionitis is a complex condition and clinical signs and symptoms are not always accurate enough for diagnosing it. It is apparent that midwives’ insufficient knowledge results in incorrect practices regarding screening for and managing chorioamnionitis. Worldwide it is accepted that professional development bridges the gap between knowledge and evidence-based practice (Greenaway et al. 2019). There is an urgency to make concerted efforts to upscale the in-service training programmes to assist midwives in gaining specific knowledge related to conditions such as chorioamnionitis for better screening and management practices.

Midwives in this study expressed that there is a lack of screening for chorioamnionitis in antenatal clinics in the setting and revealed that there are no screening tools available for chorioamnionitis; screening for UTIs and vaginal infections are not linked to chorioamnionitis and treated inconsistently. For consistent and best practices in the screening and management of chorioamnionitis, it is therefore important to have these screening tools available as well as a linked treatment plan (American College of Obstetricians and Gynecologists 2017).

Because chorioamnionitis is an infection, it shares common clinical signs and symptoms such as an increased pulse rate, increased foetal heart rate, abdominal tenderness with a closed os, leukocytes in the urine and increase maternal temperature (Committee on Obstetric Practice 2017) with other infections, such as pyelonephritis or UTIs. Thus, the diagnosis is often missed for the woman at risk for chorioamnionitis, or signs and symptoms of chorioamnionitis is undiagnosed. This was confirmed by the National Institutes of Health (United States of America) who contributed this to the broad definition of chorioamnionitis, which refers to a heterogeneous group of various conditions including inflammation as well as infections of varying degrees of severity and duration (Higgins et al. 2016).

Participants reported that there is no different management during labour, birth and the postpartum period for women who were exposed to chorioamnionitis. There are no specific guidelines regarding treatment and follow-up of women with chorioamnionitis after birth. After birth, antimicrobials prescribed for women with chorioamnionitis are continued depending on the clinical condition of the woman, especially when there is ongoing fever (Committee on Obstetric Practice 2017).

Furthermore, Chapman et al. (2014) report that some studies supported longer than 6 weeks of postnatal follow-up but that, most importantly, it is necessary to check for additional symptoms of infection or relapse, defined as ‘a cure with subsequent wound infection, abscess or recurrent endometritis’. This further necessitates best practice guidelines for screening and managing chorioamnionitis not only to prevent postnatal maternal complications but also to prevent and detect chorioamnionitis in subsequent pregnancies, which could cause maternal and neonatal complications. However, as this research indicates, midwives do not use the current guidelines available to them. Midwives indicated they were not up-to-date with the guidelines, which could result in midwives neglecting to integrate theory and practice and to use the guidelines to guide their practice, which was indicated elsewhere as one of the common barriers to translate knowledge into practice (Ausmed 2018). Midwives also indicated to be demotivated to use the guidelines because doctors attached little value to the guidelines for chorioamnionitis in the *Guidelines for Maternity Care in South Africa* (South Africa National Department of Health 2015). This may have led to midwives incorrectly perceiving that the guidelines are not important or applicable or that the guidelines are not effective, because medical practitioners override the guidelines when consulted telephonically.

Guidelines should reflect evidence-based guidance for clinical decision-making and practice. When midwives forsake to pay attention to guidelines or omit to practice according to guidelines, it poses a risk for clinical decision-making errors, which could disadvantage the patient and health outcomes. It is not well understood why clinical practitioners do not follow guidelines. An analyst, Etienne (2010) reckoned that human responses to following rules could be an aspect of automatic behaviour – the product of habits and routines. Rycroft-Malone et al. (2010) assert that, in practice, practitioners are rarely bound to follow guidelines, protocols and standards.

Filipe et al. (2014), however, concluded that continuous educational development was identified as a major factor that influences clinical decision-making practice between nurses and midwives.

At a *Maternal Care Quality Standards* workshop that was held in Pretoria in April 2016 (Maternal Care Quality Standards in the South African Context 2016), it was said that it is expected that adherence to the guidelines would significantly influence maternal and new-born outcomes. Internationally, there seems to be more evidence-based guidance. For example, the American College of Obstetricians and Gynaecologists developed a guideline on Intrapartum Management of Chorioamnionitis, including intrapartum treatment of chorioamnionitis as well as postpartum and neonatal care (Wisner 2018).

In summary, a combination of factors in practice influences midwives’ lack of knowledge regarding chorioamnionitis, namely attitudes to professional development and in-service training and conflict between the guidelines and instructions from medical practitioners.

## Limitations of the study

The first author’s presence during data gathering, which is unavoidable in qualitative research, could have affected the subjects’ responses in terms of the provided scenario as well as their general perceptions regarding the midwifery practices related to the screening and management of women who are at risk of or diagnosed with chorioamnionitis. The choice of research methodology could have been a possible limitation in so far as the semi-structured interviews might have sacrificed depth, allowing the possibility of superficial discourse. The use of a scenario for a certain group of participants may have unintentionally aided the perception that the research was seeking to apportion blame.

## Conclusion

Findings of this research showed that midwives lacked knowledge regarding the screening and management of women with chorioamnionitis resulting in incorrect practices in this regard. Further, midwives in this study neglect to update their professional knowledge on the recommended midwifery practices related to the screening and management of women who are at risk of or diagnosed with chorioamnionitis updating their knowledge is thus a recommendation.

A literature review related to maternal mortality rates showed that training in essential management of obstetric emergencies could be effective, but needs the support of concerted efforts to upscale the in-service training programmes related to midwifery, obstetrics and neonatal care (Moran, Naidoo & Moodley 2015). With the implementation of the requirement for continuous development points for annual re-registration with the South African Nursing Council underway, universities and nursing colleges should focus on short learning programmes that include training on the diagnosis and management of chorioamnionitis. Findings of this study are applicable to midwifery practice, midwifery education and midwifery research.
